# Knowledge and practices regarding postoperative neonatal pain assessment among healthcare professionals in Kinshasa: a multicenter cross-sectional survey

**DOI:** 10.3389/fpain.2026.1968968

**Published:** 2026-10-07

**Authors:** Eriksson Landu, Joseph Nsiala, Arriel Makembi Bunkete, Wilfrid Mbombo, Stéphane Mutombo, Patrick Mukuna, Carl Lemba, Christian Nantulu, Patricia Kabuni, Alphonse Mosolo, Jean-Pierre Ilunga, Benjamin Kabwe, Médard Bulabula, Berthe Barhayiga

**Affiliations:** 1 Department of Anesthesiology and Critical Care, University of Kinshasa, Kinshasa, Democratic Republic of the Congo; 2 Department of Anesthesiology and Critical Care, Necker -Enfants Malades University Hospital, AP-HP, Sorbonne University, Paris, France; 3 Department of Anesthesiology and Critical Care, Ngaliema Medical Center, Kinshasa, Democratic Republic of the Congo; 4 Department of Anesthesiology and Critical Care, Hôpital Privé de Thiais, Thiais, France; 5 Department of Internal Medicine and Nephrology, University of Kinshasa, Kinshasa, Democratic Republic of the Congo; 6 Department of Anesthesiology and Critical Care, Monkole Hospital Center, Kinshasa, Democratic Republic of the Congo; 7 Department of Anesthesiology and Critical Care, Robert Debré University Hospital, AP-HP, Paris, Democratic Republic of the Congo

**Keywords:** democratic Republic of Congo, healthcare professionals, Kinshasa, knowledge, neonatal pain, pain assessment, postoperative pain

## Abstract

**Background:**

Neonatal pain remains inconsistently assessed, particularly in resource-limited settings. We assessed healthcare professionals’ knowledge and reported practices regarding postoperative neonatal pain assessment in Kinshasa, Democratic Republic of the Congo (DRC).

**Methods:**

We conducted a multicenter cross-sectional survey from August 2025 to May 2026 in ten hospitals with pediatric surgical activity. Healthcare professionals completed a 20-item questionnaire developed through expert content review and pretesting. The primary outcome was self-reported use of a standardized neonatal pain-assessment tool. A four-item knowledge score (0–4) was constructed, with ≥3 indicating high knowledge as a pragmatic study-specific threshold. Multivariable logistic regression used hospital-level cluster-robust standard errors.

**Results:**

Of 580 healthcare professionals approached, 536 returned the questionnaire; six were excluded because of >30% missing data, leaving 530 complete questionnaires for analysis. Although 86.8% recognized neonatal pain, only 10.0% reported using a standardized pain-assessment tool, and 15.3% reported postoperative pain assessment at regular intervals. Nearly all participants (99.8%) reported no dedicated neonatal pain training. The mean knowledge score was 2.16 ± 0.84, and 34.7% had high knowledge. Higher knowledge was independently associated with reported standardized tool use (adjusted OR 4.90, 95% CI 3.10–7.70; *p* < 0.001). In the prespecified sensitivity analysis using the reduced three-item knowledge score, the association remained significant but was attenuated (adjusted OR 1.93, 95% CI 1.23–3.03; *p* = 0.004).

**Conclusion:**

Reported use of standardized neonatal pain-assessment tools and regular postoperative pain assessment were low despite widespread recognition of neonatal pain. Higher knowledge was associated with reported tool use, although the association was attenuated after excluding scale recognition from the knowledge score. These findings highlight gaps in neonatal pain assessment and training, while the cross-sectional design precludes causal interpretation.

## 1 Introduction

Neonatal pain was long overlooked in clinical practice, partly because of historical uncertainty regarding the newborn infant's capacity to perceive, express, and respond to pain, including the belief that cortical immaturity limited nociceptive processing (1, 2). Subsequent clinical and neurophysiological evidence demonstrated that newborn and preterm infants are capable of processing noxious stimuli (2, 3). In addition, repeated exposure to untreated pain during the neonatal period may have short- and long-term effects on neurodevelopment and subsequent pain processing (4, 5).

The recognition of neonatal pain has therefore become an important component of high-quality neonatal care. Accurate assessment is essential because newborn infants cannot verbally report pain and clinicians must rely on behavioural and physiological indicators. Several clinical rating scales have been developed for neonatal pain assessment. These instruments differ in their intended populations and clinical contexts, including acute procedural pain, prolonged pain, and postoperative pain. Recent systematic evidence has identified a broad range of neonatal pain-assessment scales and raised concerns regarding the quality of evidence supporting their validity, reliability, and applicability across neonatal populations and clinical settings (6). Consequently, the choice of a pain-assessment instrument should consider the population, type of pain, and clinical context for which it has been evaluated (6).

Neonatal pain occurs in several contexts, including repeated painful procedures and postoperative care. Surgical procedures may expose neonates to substantial acute and ongoing pain, making appropriate assessment particularly important. Non-pharmacological interventions, including breastfeeding, skin-to-skin care, facilitated tucking, and other supportive approaches, may reduce procedural pain, although their effectiveness and applicability vary according to the intervention and clinical context (7–9). These approaches should therefore be considered within the broader framework of neonatal pain assessment and management rather than assumed to be equivalent to postoperative analgesia.

Despite increasing recognition of neonatal pain, implementation of standardized pain assessment remains inconsistent. Reviews have highlighted substantial heterogeneity in the use and reporting of neonatal pain scales, differences in their measurement properties, and the importance of selecting instruments appropriate to the neonatal population and pain context (10–12). In sub-Saharan Africa, available evidence indicates persistent gaps in pediatric pain assessment and management, while research remains limited and heterogeneous across populations and clinical settings (13).

In the Democratic Republic of the Congo (DRC), previous work has documented neonatal pain associated with clinical procedures, including assessment using the Douleur Aiguë du Nouveau-né (DAN) scale (14). However, to the best of our knowledge, no previous multicenter study has assessed healthcare professionals' knowledge and reported practices regarding postoperative neonatal pain assessment in Kinshasa.

This study aimed to assess knowledge and reported practices regarding postoperative neonatal pain assessment among healthcare professionals in Kinshasa and to identify factors independently associated with the reported use of a validated neonatal pain-assessment scale.

## 2 Methods

### 2.1 Study design and setting

We conducted a multicenter cross-sectional survey from August 27, 2025, to May 20, 2026, in Kinshasa, Democratic Republic of the Congo (DRC). The study included the ten hospitals providing pediatric surgical activity in Kinshasa, comprising three tertiary-level and seven secondary-level hospitals. These ten hospitals represented the exhaustive set of hospitals providing pediatric surgical activity in Kinshasa during the study period and were therefore all approached for participation. The study aimed to assess healthcare professionals' knowledge and reported practices regarding postoperative neonatal pain assessment and to identify factors associated with reported use of a validated neonatal pain assessment scale.

### 2.2 Participants and eligibility criteria

Healthcare professionals involved in the perioperative care of neonates were eligible to participate. Eligible participants included nurses and other paramedical staff, nursing students, general practitioners, medical trainees, interns and residents, and medical specialists. Medical specialists included pediatricians, anesthesiologists/intensivists, and surgeons. Participants were eligible if they were working in one of the participating hospitals and were involved in the perioperative or postoperative care of neonates. To ensure sufficient exposure to the clinical environment and basic experience in perioperative neonatal care, eligibility required at least six months of professional seniority in the concerned department. This six-month period corresponds to the regulatory probationary period in the Congolese administration, during which personnel are expected to demonstrate their ability to perform the basic duties of their position. Participation was voluntary and required informed consent.

### 2.3 Questionnaire development and data collection

Data were collected using a 20-item self-administered questionnaire developed specifically for this study. The questionnaire was developed in French based on the study objectives and covered professional characteristics, knowledge of neonatal pain, pain-assessment practices, pharmacological and non-pharmacological pain-management practices, and perceived barriers to neonatal pain assessment.

The questionnaire was evaluated by 15 independent experts in pediatrics and public health for content validity. Each item was assessed for clarity, relevance, and appropriateness. The item-level content validity index (I-CVI) was calculated, with an I-CVI of ≥0.78 considered acceptable. The scale-level content validity index based on the average method (S-CVI/Ave) was also calculated, with a value of ≥0.90 considered acceptable. A pretest was subsequently conducted among 15 healthcare professionals to assess clarity and comprehension, and minor wording adjustments were made before the main survey.

Questionnaires were administered individually by trained local investigators at each participating hospital. Each questionnaire was assigned a unique code and was handed directly to one participant, thereby limiting the possibility of multiple questionnaires being attributed to the same participant. The questionnaire was completed anonymously, independently, and without consultation with colleagues. Participants completed the questionnaire during the middle of the day on a non-operative working day, to minimize interruptions related to surgical activity, and returned the completed questionnaire 24 h later. No manager, physician, head nurse, or other supervisor was involved in the completion of the questionnaire. No identifying information was collected.

Because the questionnaire assessed reported practices, all practice-related outcomes were based on participants' self-reports and were not independently verified by direct observation or medical-record review. The complete English version of the questionnaire is provided as (Supplementary File S1).

### 2.4 Data management and quality control

Completed questionnaires were entered independently by two trained operators. The two databases were compared, and discrepancies were checked against the original questionnaires and resolved before analysis. Of the 536 returned questionnaires, six were excluded because more than 30% of the questionnaire data were missing. The remaining 530 questionnaires contained no missing responses and were therefore complete and included in the final analysis. No imputation of missing data was required.

### 2.5 Study variables and operational definitions

#### 2.5.1 Primary outcome

The primary outcome was reported use of a validated neonatal pain assessment scale, based on the questionnaire item “Do you use a standardized pain assessment tool?”, with response options “Yes” and “No”. For analysis, a “Yes” response was considered reported use, whereas a “No” response was considered non-use. Because the questionnaire used a binary response, the primary outcome captured whether participants reported using a pain-assessment scale and did not quantify the frequency or regularity of use. The primary outcome therefore reflects self-reported practice and was not independently verified through direct observation or medical-record review.

For this study, a validated neonatal pain-assessment scale was defined *a priori* as an instrument with published evidence of validation in neonatal populations and relevant to the assessment of neonatal pain. Evidence of validation in broader pediatric populations alone was considered insufficient. However, validation in a neonatal population was not considered equivalent to specific validation for postoperative neonatal pain, because neonatal pain-assessment instruments have been developed and validated for different pain contexts. The predefined list included NIPS, EDIN, EVENDOL, and DAN. NIPS and DAN were primarily validated for acute procedural pain, EDIN for prolonged pain in preterm infants, and EVENDOL has supporting validation evidence in neonatal populations for procedural and acute non-procedural pain. None of these instruments was classified in the present study on the basis of specific validation for postoperative neonatal pain. A predefined list of neonatal pain-assessment scales meeting the study criteria was established before data analysis based on the available literature and was used to classify participants' reported scale use. Accordingly, recognition or reported use of these instruments was interpreted as recognition or reported use of a validated neonatal pain-assessment scale, rather than as evidence of recognition or use of an instrument specifically validated for postoperative neonatal pain. Recognition of a validated neonatal pain-assessment scale was assessed separately as a knowledge item.

The recognition of a validated neonatal pain-assessment scale was assessed as one component of the study-specific knowledge score. This item was distinct from the primary outcome in that it assessed knowledge/recognition, whereas the primary outcome assessed reported use. Nevertheless, the conceptual overlap between the two variables was considered in the analysis and addressed through a sensitivity analysis excluding the scale-recognition item from the knowledge score.

#### 2.5.2 Regular postoperative pain assessment

Regular postoperative pain assessment was assessed using the questionnaire item “How often do you assess pain after surgery in a newborn?” Response options included “at postoperative admission only,” “according to the clinical condition,” “at regular intervals (e.g., every 4 h),” and “never.” Multiple responses were possible. For analysis, regular postoperative pain assessment was defined as selection of “at regular intervals (e.g., every 4 h),” either alone or in combination with another response option.

#### 2.5.3 Non-pharmacological methods

Non-pharmacological methods were defined as self-reported use of at least one non-pharmacological intervention specifically in the context of postoperative neonatal care. This threshold was used to classify whether any non-pharmacological postoperative pain-management practice was reported. The interventions assessed in the questionnaire included breastfeeding, skin-to-skin contact, pacifier use, and containment. Each intervention was recorded separately when multiple responses were permitted.

Breastfeeding and skin-to-skin contact were considered in this study as reported supportive/analgesic measures used in the postoperative neonatal context, rather than as indicators of general neonatal comfort outside the postoperative setting.

#### 2.5.4 Analgesic practice

Analgesic practice was assessed as self-reported use of analgesic medications listed in the questionnaire. The questionnaire included paracetamol, ibuprofen, opioids, and other reported analgesic options. Because the questionnaire did not systematically collect dose, route, timing, gestational age, postnatal age, indication, or contraindications, reported medication use was not classified as pharmacologically “appropriate” or “good practice.” The analysis therefore describes reported analgesic use rather than the clinical appropriateness of the prescribed regimen.

#### 2.5.5 Knowledge score

Knowledge was assessed using four predefined dichotomous items addressing key aspects of neonatal pain recognition and assessment: (1) recognition that neonates experience pain; (2) recognition that the neonatal nociceptive system is partially developed but functionally capable of transmitting pain; (3) recognition of at least three key clinical signs of neonatal pain; and (4) recognition of at least one validated neonatal pain-assessment scale.

Each item was scored as 1 for a correct response and 0 for an incorrect response or a “don't know” response. Missing responses were retained as missing. The four item scores were summed to obtain a total knowledge score ranging from 0 to 4. For descriptive purposes, a score of ≥3 was considered relatively high knowledge. This threshold was pragmatic and study-specific and was not based on an externally validated or empirically established cutoff.

The four items were considered distinct but related domains of neonatal pain knowledge rather than indicators of a single formally validated latent construct. Internal consistency was therefore assessed descriptively using Cronbach's alpha rather than as evidence of unidimensionality or construct validity. Because all four items were dichotomous and scored 0/1, Cronbach's alpha is mathematically equivalent to the Kuder–Richardson 20 (KR-20) coefficient under standard scoring. The resulting coefficient was interpreted only as a descriptive indicator of internal consistency; it was not used to establish the validity of the knowledge score. A modest internal-consistency coefficient would not, by itself, imply poor item quality because the items covered different knowledge domains.

Because recognition of a validated pain-assessment scale was conceptually related to the primary outcome of reported use of a standardized pain-assessment tool, a reduced three-item knowledge score was also constructed for sensitivity analysis. This score excluded the scale-recognition item and retained the same scoring approach, with a possible range from 0 to 3.

#### 2.5.6 Good-practice score

Reported good practice was summarized using four predefined postoperative pain-practice indicators derived from the questionnaire: (1) reported use of a standardized pain-assessment tool; (2) reported postoperative pain assessment at regular intervals (e.g., every 4 h); (3) reported use of at least one non-pharmacological pain-relief intervention; and (4) reported analgesic use according to the questionnaire-defined medication category.

Each indicator contributed one point, resulting in a total score ranging from 0 to 4. The four indicators were equally weighted, and a score of ≥3 defined good reported practice. Both the equal weighting and the ≥3 threshold were pragmatic, study-specific choices and were not based on an externally validated practice-quality instrument or clinical cutoff. The score was intended to summarize the number of predefined practice indicators reported by each participant; therefore, equal weighting does not imply equivalent clinical importance, effectiveness, or priority, and a score of ≥3 does not imply that all recommended aspects of postoperative neonatal pain management were appropriately performed.

For the analgesic component, the score reflected reported medication use only. Because the questionnaire did not systematically capture dose, route, timing, indication, gestational age, postnatal age, or contraindications, analgesic use was not classified as pharmacologically appropriate or interpreted as evidence of appropriate analgesic prescribing. Because use of a standardized pain-assessment tool was one of the four score components and was also the primary outcome of the main model, this created partial overlap between the composite practice outcome and the primary outcome and was considered when interpreting the findings.

For all questionnaire items, multiple responses were retained when applicable, and each predefined indicator was evaluated separately. “Don't know” responses were not considered affirmative evidence of the corresponding practice. Participants with more than 30% missing questionnaire responses were excluded from the analytic sample, as prespecified. The 530 questionnaires retained for analysis contained no missing responses; therefore, no imputation was required.

### 2.6 Statistical analysis

Categorical variables were summarized as frequencies and percentages, and continuous variables as means with standard deviations or medians with interquartile ranges, as appropriate. Proportions were reported with 95% confidence intervals. For questions allowing multiple responses, each response option was analyzed separately, and percentages were therefore not expected to sum to 100%. All analyses were conducted among the 530 participants with complete questionnaire data, after exclusion of six questionnaires with more than 30% missing responses. No imputation was required.

Multivariable logistic regression was used to identify factors associated with the prespecified neonatal pain-management outcomes. Three logistic regression models were specified *a priori*, without stepwise variable selection. Model 1 evaluated factors associated with reported use of a validated neonatal pain-assessment scale and included the four-item knowledge score, professional category, and professional experience. Model 2 evaluated factors associated with good reported practice. Model 3 evaluated factors associated with high knowledge and included professional category and professional experience.

For Model 1, the dependent variable was reported use of a validated neonatal pain-assessment scale, coded as a binary outcome (yes/no). The four-item knowledge score was entered as a continuous predictor, together with professional category and professional experience. In a prespecified sensitivity analysis of Model 1, the four-item knowledge score was replaced by the reduced three-item score excluding recognition of a validated neonatal pain-assessment scale. The sensitivity model retained the same outcome, covariates, and hospital-level cluster-robust standard errors as the primary Model 1.

For Model 2, the dependent variable was good reported practice, defined as a good-practice score of ≥3. The model included the reduced three-item knowledge score (range, 0–3), professional category (five levels; Nurse as reference), seniority (four levels; <2 years as reference), and institution (ten levels; Hospital 10 as reference). All variables were entered simultaneously without stepwise selection. The model therefore estimated 18 parameters: the intercept, one parameter for the knowledge score, four professional-category indicators, three seniority indicators, and nine institution indicators. There were 89 participants classified as having good reported practice among the 530 participants included in the analysis, corresponding to 4.9 events per estimated parameter. Hospital-level cluster-robust standard errors were used to account for potential within-hospital correlation.

Model 3 evaluated factors associated with high knowledge, defined as a four-item knowledge score of ≥3. Professional category and professional experience were entered as explanatory variables.

Institutional patterns were explored using principal component analysis (PCA) and hierarchical clustering based on ten standardized performance indicators. Because only ten institutions were available for institutional-level analysis, these analyses were considered exploratory and hypothesis-generating and were not used as confirmatory inferential analyses.

For logistic regression models, adjusted odds ratios (aORs) with 95% confidence intervals and two-sided *p*-values were reported. Model discrimination was assessed using the area under the receiver operating characteristic curve (AUC), and calibration was assessed using the Hosmer–Lemeshow goodness-of-fit test. Multicollinearity was assessed using the variance inflation factor. Hospital-level cluster-robust standard errors were used to account for the clustering of participants within hospitals. Statistical tests were two-sided, and *p* < 0.05 was considered statistically significant.

### 2.7 Potential sources of bias

Because the study relied on self-administered questionnaires, reported practices may have been affected by recall and social-desirability bias and may not fully reflect actual bedside practice. Misclassification is also possible, particularly for reported use of pain-assessment tools, because respondents may have reported a tool that they considered standardized or validated, whereas its neonatal validation status was determined according to the predefined study criteria. The study-specific questionnaire was pre-tested for clarity and comprehension but was not formally validated; therefore, measurement error cannot be excluded. Non-response bias cannot be completely excluded despite the high overall response rate. Although hospital-level cluster-robust standard errors were used, the relatively small number of participating hospitals may limit the precision of variance estimates and does not eliminate the possibility of residual institutional heterogeneity.

### 2.8 Ethical considerations

The study protocol was approved by the Ethics Committee of the School of Public Health, University of Kinshasa (approval No. ESP/CE/141B/2025). All participants provided written informed consent before participation. The study was conducted in accordance with applicable ethical principles for research involving human participants.

## 3 Results

### 3.1 Participants' characteristics

Of the 580 healthcare professionals approached, 536 completed the questionnaire (response rate, 92.4%). Six questionnaires were excluded because more than 30% of questionnaire data were missing. The remaining 530 questionnaires contained no missing responses and were included in the final analysis. The 530 participants were distributed across the ten participating hospitals, with the proportion of participants ranging from 3.3% in Hospital 10 to 21.3% in Hospital 1. Hospitals 2, 3, and 4 contributed 15.5%, 12.3%, and 12.3% of participants, respectively, while Hospitals 5, 6, 7, 8, and 9 contributed 9.4%, 8.5%, 7.4%, 5.5%, and 4.5%, respectively.

Nurses were the largest professional group (*n* = 114; 21.5%), followed by general practitioners (*n* = 104; 19.6%) and medical trainees (*n* = 78; 14.7%). Medical specialists comprised pediatricians, surgeons, anesthesiology/ICU professionals, and other specialist categories reported in the questionnaire. Pediatricians, surgeons, anesthesiology/ICU professionals, residents, and anesthesia technicians accounted for 10.2%, 6.2%, 6.0%, 6.0%, and 4.5% of participants, respectively.

Overall, 130 participants (24.5%) reported knowing at least one pain-assessment scale, and 53 (10.0%) reported using a standardized neonatal pain-assessment tool. The distribution of professional functions and selected knowledge and reported-practice indicators is shown in Figure 1.

**Figure 1:**
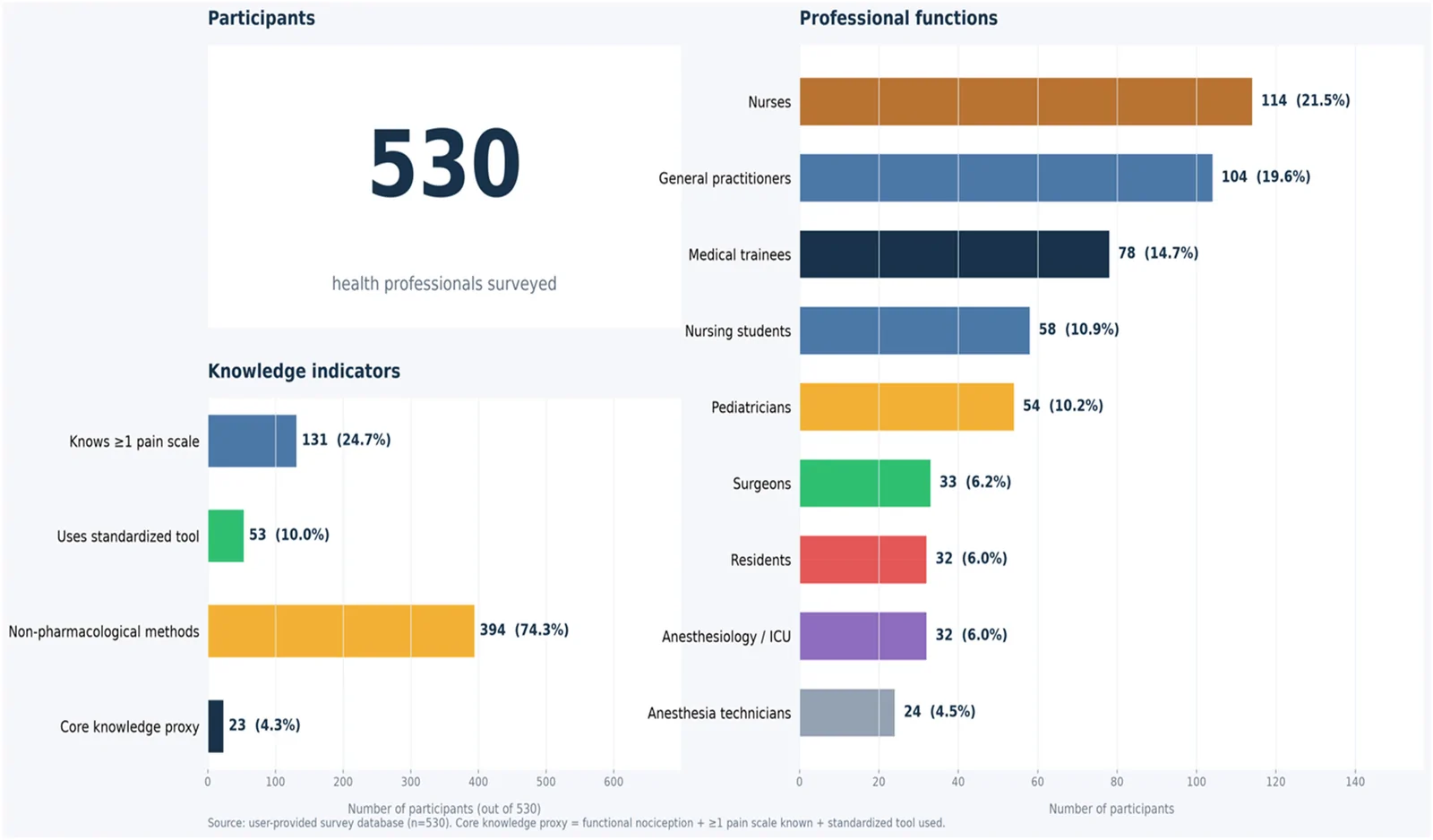
Professional characteristics and selected neonatal pain knowledge and practice indicators among participants.

### 3.2 Recognition of neonatal pain and reported assessment practices

High-pitched continuous crying was the most frequently recognized clinical sign of neonatal pain (81.7%), followed by changes in vital signs and withdrawal movements or agitation (61.1% each), while facial expressions or grimacing were recognized by 55.1% of participants. Knowledge of neonatal pain-assessment scales was limited, with 400 participants (75.5%) reporting that they knew no scale and 130 (24.5%) reporting knowledge of at least one scale. Multiple responses were permitted for the scale-knowledge item; therefore, individual scale frequencies were not mutually exclusive and percentages were not expected to sum to 100%.

Overall, 53 participants (10.0%) reported using a standardized pain-assessment tool. Among these 53 participants, the scales reported included DAN in 33 (62.3%), NIPS in 21 (39.6%), EDIN in 13 (24.5%), PIPP in 10 (18.9%), EVENDOL in 9 (17.0%), NFCS in 8 (15.1%), and Comfort/Comfort Behavior in 7 (13.2%). Participants could report more than one scale; therefore, the scale-specific categories overlapped, were not mutually exclusive, and their percentages were not expected to sum to 100%. All scale-specific percentages were calculated using the 53 participants reporting standardized tool use as the denominator. Because tool use was assessed using a binary self-reported response, these figures represent reported use and do not indicate the frequency or regularity of use and were not objectively verified.

Regarding the frequency of postoperative pain assessment, 81 participants (15.3%) reported assessing neonatal pain at regular intervals (e.g., every 4 h), either alone or in combination with other response options. Of these, 61 participants (11.5%) selected regular-interval assessment as their sole response. In contrast, 40 participants (7.5%) reported never assessing postoperative neonatal pain (Figure 2).

**Figure 2:**
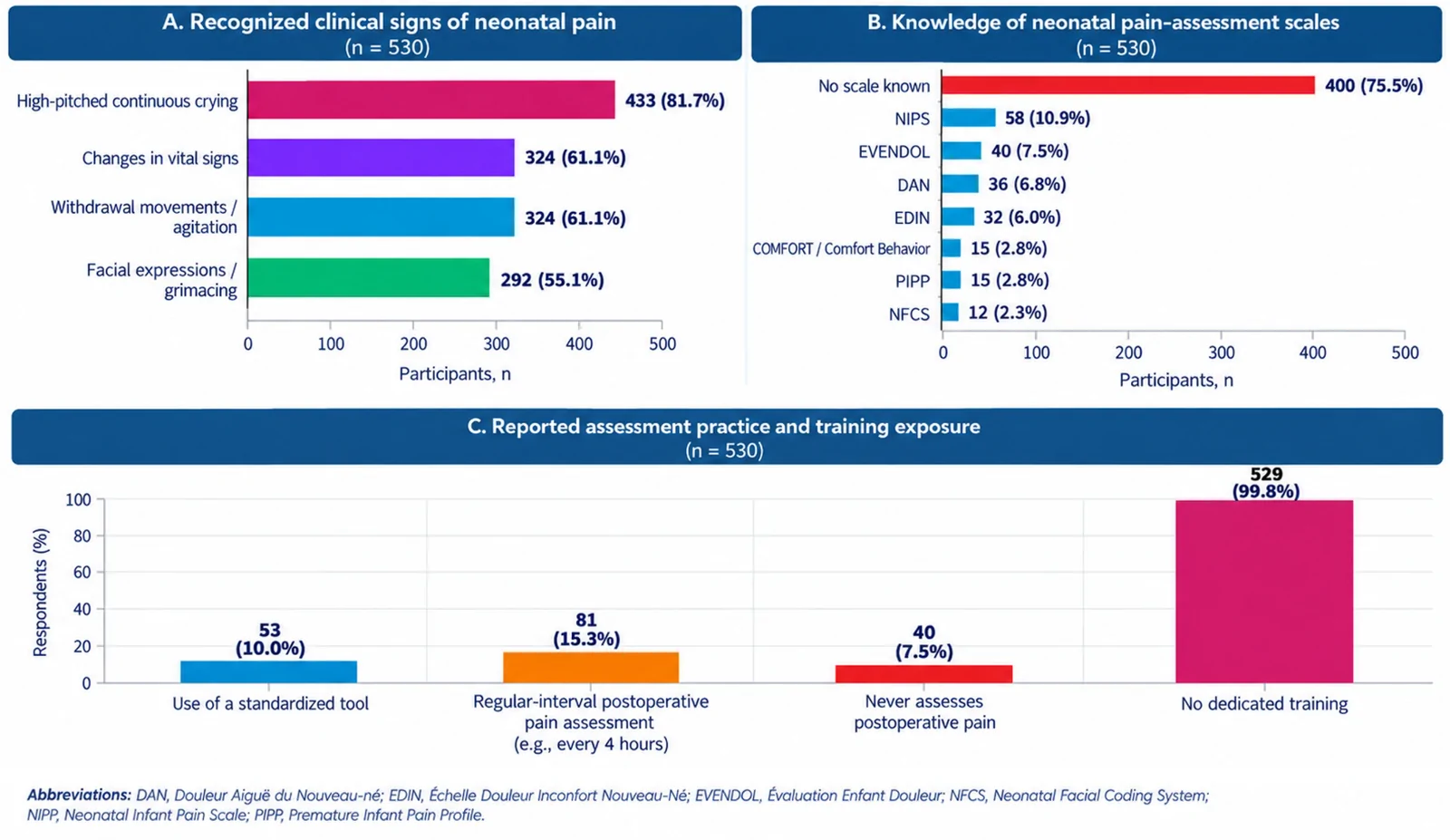
Recognition of neonatal pain, knowledge of pain-assessment scales, and reported assessment practices among healthcare professionals (*n* = 530). Values are presented as number (percentage). Multiple responses were permitted for pain-assessment scales; therefore, percentages do not sum to 100%.

### 3.3 Reported analgesic and non-pharmacological practices

Paracetamol was the most frequently reported analgesic (89.4%), followed by ibuprofen (43.6%). Reported opioid use included morphine (23.4%), fentanyl (18.5%), and tramadol (5.1%). Non-pharmacological pain-management methods were reported by 394 participants (74.3%). All 530 participants reported using at least one pharmacological analgesic. Of these, 394 (74.3%) also reported using non-pharmacological methods, whereas 136 (25.7%) reported pharmacological approaches only. Consequently, no participants were classified as using non-pharmacological methods only or neither approach. Participants could report more than one analgesic or non-pharmacological intervention; therefore, percentages for individual interventions were not mutually exclusive. These findings describe self-reported medication use and do not establish the pharmacological appropriateness of the reported regimens, because dose, route, timing, indication, gestational age, postnatal age, and contraindications were not systematically assessed (Table 1).

**Table 1:** Knowledge and reported good-practice indicators and score distributions among participants.

Measure	n (%)/Mean ± SD
Reported practice indicators	
Reported analgesic use	530 (100.0%)
Use of non-pharmacological methods	394 (74.3%)
Regular-interval postoperative pain assessment	81 (15.3%)
Use of a standardized neonatal pain-assessment tool	53 (10.0%)
Knowledge score (0–4)	
Mean ± SD	2.16 ± 0.84
Median (IQR)	2 (2–3)
High knowledge (≥3)	184 (34.7%)
Good-practice score (0–4)	
Mean ± SD	1.42 ± 0.96
Median (IQR)	1 (1–2)
Good practice (≥3)	89 (16.8%)

IQR, interquartile range; SD, standard deviation.

### 3.4 Knowledge and good-practice scores: exploratory assessment and distribution

The four-item knowledge score had a mean of 2.16 ± 0.84 and a median of 2 (IQR: 2–3). A total of 184 participants (34.7%) had a score ≥3 and were therefore classified as having relatively high knowledge according to the predefined study-specific threshold. The four knowledge items covered distinct but related domains of neonatal pain knowledge. Each correct response was scored as 1, whereas incorrect and “don't know” responses were scored as 0; missing responses were retained as missing. Internal consistency was assessed descriptively using Cronbach's alpha, which was 0.71. Because the items were dichotomous, this value is mathematically equivalent to the KR-20 coefficient under standard scoring. The coefficient was interpreted descriptively and was not considered evidence of unidimensionality or construct validity, given the distinct domains covered by the four items. A reduced three-item knowledge score excluding the item on recognition of a validated neonatal pain-assessment scale was also used for sensitivity analysis because of its conceptual overlap with the primary outcome. This reduced score was used to assess whether the association between knowledge and reported use of a standardized pain-assessment tool remained after removing the potentially overlapping item. The good-practice score had a mean of 1.42 ± 0.96 and a median of 1 (IQR: 1–2). Eighty-nine participants (16.8%) met the predefined threshold of ≥3 indicators and were classified as having good reported practice (Table 1).

### 3.5 Factors associated with neonatal pain-assessment knowledge and reported practices

In bivariable analyses, reported use of a validated neonatal pain-assessment scale was associated with professional category (*χ*^2^ = 17.915, df = 4; *p* = 0.0013), institution (*χ*^2^ = 21.520, df = 9; *p* = 0.0105), and knowledge level (*χ*^2^ = 352.491, df = 2; *p* < 0.0001). Knowledge scores differed significantly according to professional category and seniority (*p* < 0.001 and *p* = 0.007, respectively). Higher knowledge levels were also associated with greater reported use of non-pharmacological methods (*χ*^2^ = 34.268, df = 2; *p* < 0.0001). Seniority and institution were associated with good-practice scores (*p* < 0.001 and *p* = 0.034, respectively), while institution was associated with reported opioid use (*p* = 0.035) but not with non-pharmacological methods (*p* = 0.469). Detailed bivariable associations are presented in Table 2.

**Table 2:** Bivariable associations between professional characteristics, knowledge, and reported neonatal pain-management practices.

Analysis	Test	Statistic	df	*p*-value
Professional category × scale use	*χ* ^2^	17.915	4	0.0013
Institution × scale use	χ^2^	21.520	9	0.0105
Institution × knowledge score	ANOVA	1.991	9, 520	0.0384
	Kruskal–Wallis	15.013	9	0.0906
Professional category × knowledge score	ANOVA	6.334	4, 525	<0.0001
	Kruskal–Wallis	22.815	4	<0.0001
Seniority × good-practice score	χ^2^	20.268	3	<0.0001
Institution × good-practice score	χ^2^	18.132	9	0.0337
Knowledge level × scale use	χ^2^	352.491	2	<0.0001
Knowledge level × non-pharmacological methods	χ^2^	34.268	2	<0.0001
Institution × opioid use	χ^2^	18.038	9	0.0347
Institution × non-pharmacological methods	χ^2^	8.665	9	0.4687
Seniority × knowledge score	ANOVA	4.060	3, 526	0.0072
	Kruskal–Wallis	16.482	3	0.0009
Professional category × non-pharmacological methods	χ^2^	16.468	4	0.0025

χ^2^, chi-square test; ANOVA, analysis of variance; df, degrees of freedom. Values are test statistics from the indicated bivariable tests.

### 3.6 Multivariable factors associated with neonatal pain-management outcomes

Three *a priori* specified logistic regression models were fitted without stepwise selection. In Model 1, higher knowledge scores were independently associated with reported use of a standardized neonatal pain-assessment tool (aOR 4.90, 95% CI 3.10–7.70; *p* < 0.001) (Figure 3A). In the prespecified sensitivity analysis using the reduced three-item knowledge score, the association remained significant but was attenuated (aOR 1.93, 95% CI 1.23–3.03; *p* = 0.004), with lower discrimination (AUC 0.745, approximate 95% CI 0.68–0.81) and poorer calibration (Hosmer–Lemeshow *χ*^2^ = 22.4; *p* = 0.004).

**Figure 3:**
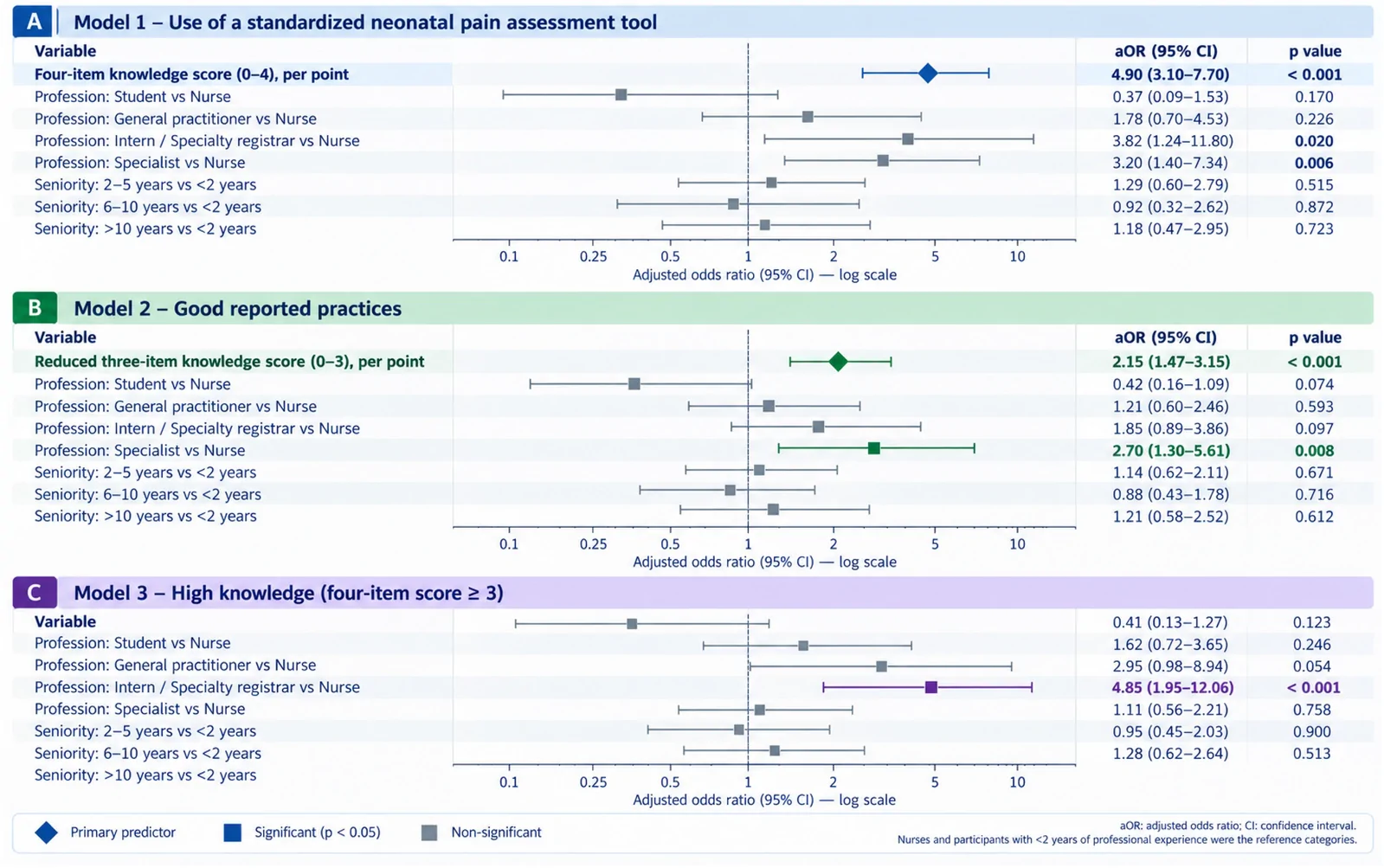
Multivariable factors independently associated with neonatal pain-management outcomes.

In Model 2, higher reduced three-item knowledge scores (aOR 2.15, 95% CI 1.47–3.15; *p* < 0.001) and medical specialist status vs. nursing staff (aOR 2.70, 95% CI 1.30–5.61; *p* = 0.008) were independently associated with good reported practices (Figure 3B). Model discrimination was acceptable (AUC 0.806), with no evidence of poor calibration (Hosmer–Lemeshow *χ*^2^ = 4.74; *p* = 0.78).

In Model 3, medical specialist status was independently associated with high knowledge (four-item knowledge score ≥3) compared with nursing staff (aOR 4.85, 95% CI 1.95–12.06; *p* < 0.001) (Figure 3C).

### 3.7 Institutional structure analyses

PCA applied to ten standardized performance indicators explained 76.6% of the variance on the first two components (PC1 = 53.1%, PC2 = 23.4%). PC1 reflected “practice maturity” (knowledge, scale use, and regular assessment), whereas PC2 contrasted non-pharmacological methods with opioid use (Figure 4). Ward hierarchical clustering identified three exploratory institutional patterns: (i) Cluster 1: Hospitals 2, 3, 6, and 8 (low knowledge, high use of non-pharmacological methods); (ii) Cluster 2: Hospitals 1, 4, and 10 (best overall profile); and (iii) Cluster 3: Hospitals 5, 7, and 9 (more frequent opioid use) (Figure 5). The optimal three-cluster solution was supported by the silhouette coefficient (0.51) and inertia elbow. Given the small number of institutions (*n* = 10), these clusters were considered exploratory rather than stable or generalizable institutional phenotypes.

**Figure 4:**
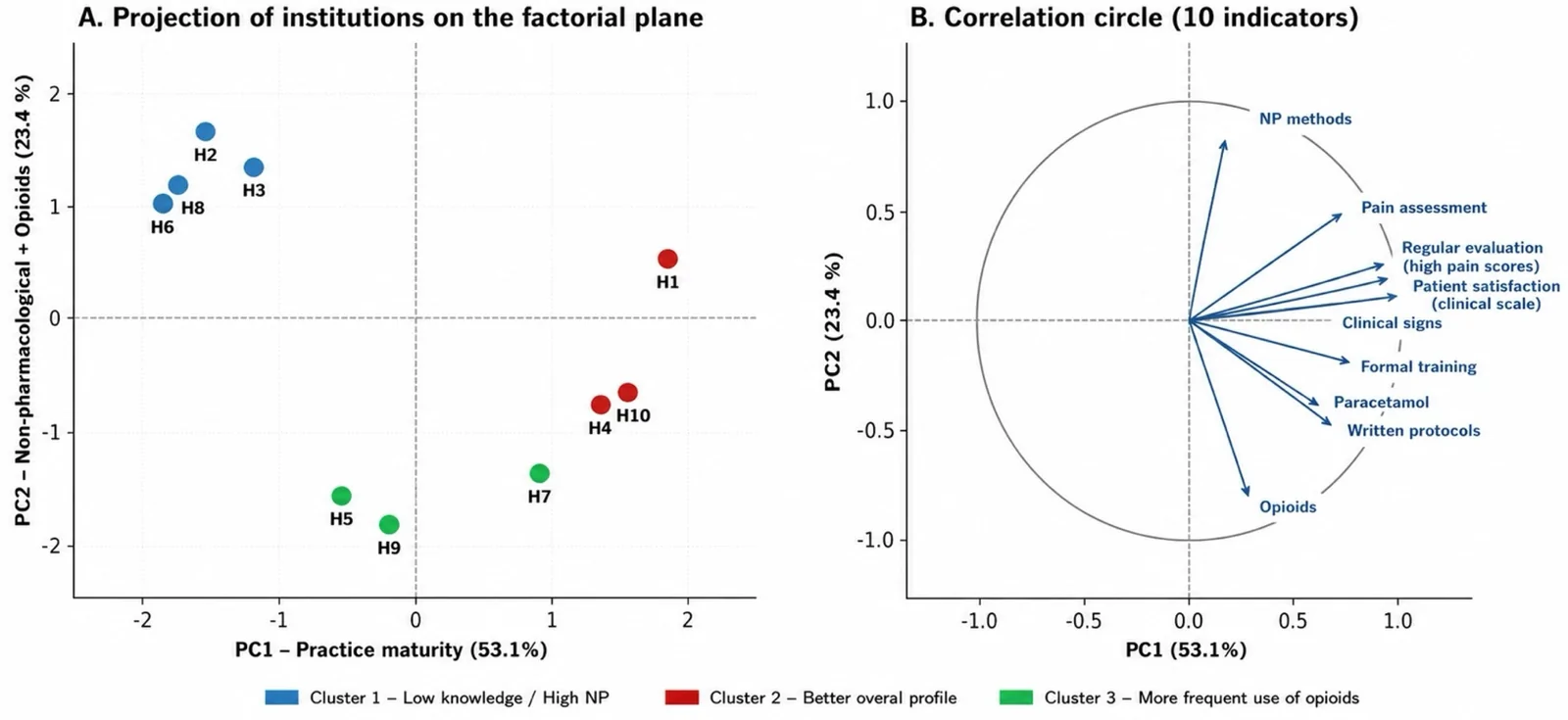
Principal component analysis of institutions and correlation circle of indicators (76.6% of variance explained).

**Figure 5:**
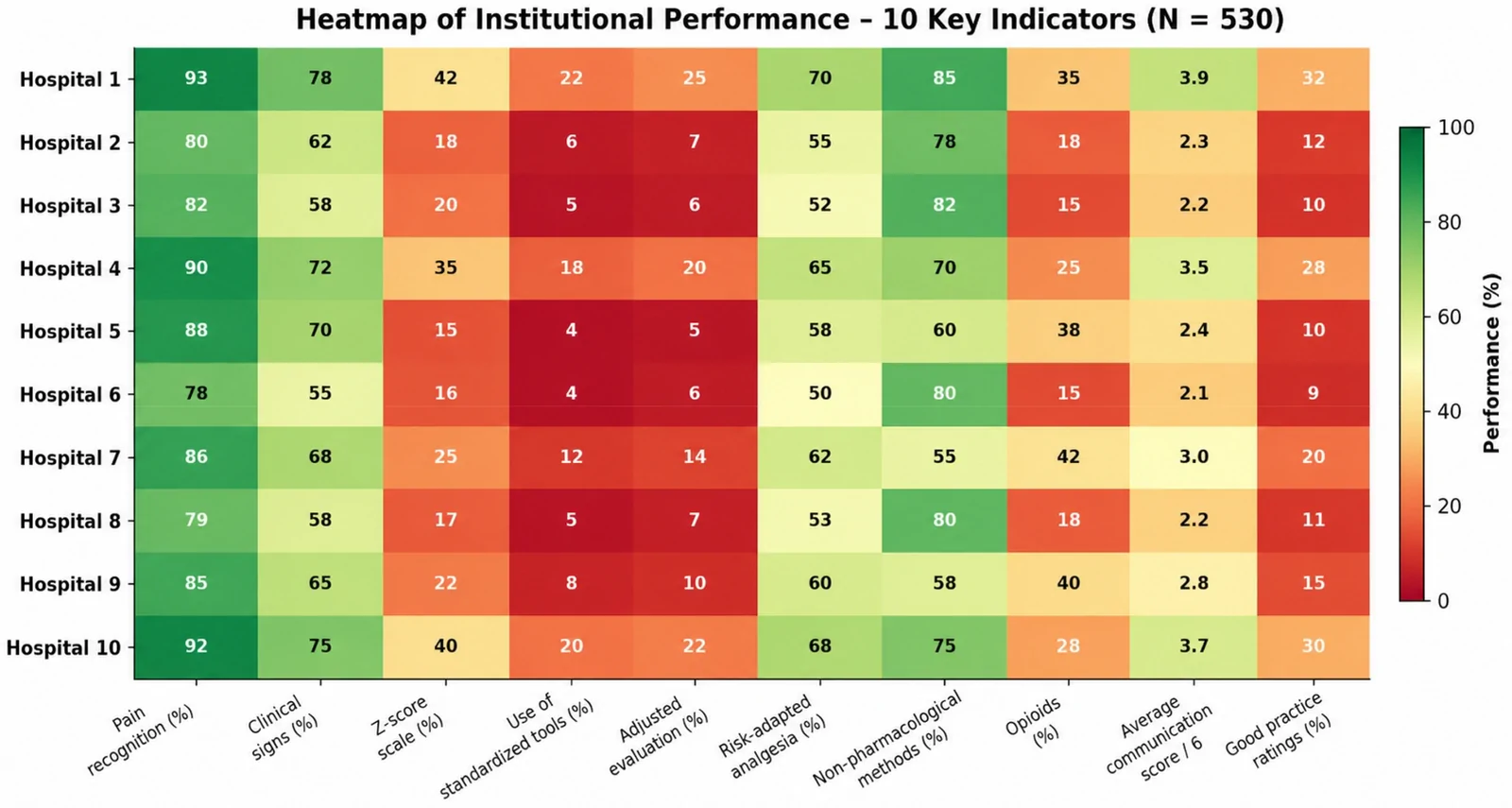
Heatmap of institutional performance: percentage of professionals fulfilling each indicator (*N* = 530). The figure is provided at full page width to improve readability of the ten-institution×ten-indicator matrix.

## 4 Discussion

This multicenter survey of 530 healthcare professionals across ten hospitals in Kinshasa identified a substantial gap between recognition of neonatal pain and reported use of validated neonatal pain-assessment scales. Although 86.8% of participants recognized neonatal pain, only 10.0% reported using a standardized neonatal pain-assessment tool, and 15.3% reported postoperative pain assessment at regular intervals. These findings are broadly consistent with reports from other sub-Saharan African settings, where recognition of neonatal pain has not consistently translated into standardized assessment and management practices (15–18). However, direct comparison of percentages should be made cautiously because the populations, healthcare settings, questionnaires, and outcome definitions differed across studies.

Knowledge of neonatal pain-assessment scales was also limited. The low recognition of DAN is notable given its previous local use and its application to neonatal procedural pain (19). Importantly, the neonatal pain-assessment scales included in the predefined classification were validated in different pain contexts and were not necessarily specifically validated for postoperative neonatal pain. NIPS and DAN were primarily validated for acute procedural pain, EDIN for prolonged pain in preterm infants, and EVENDOL has supporting validation evidence in neonatal populations for procedural and acute non-procedural pain. Therefore, the reported recognition or use of these instruments should not be interpreted as evidence of recognition or use of a scale specifically validated for postoperative neonatal pain. Rather, the 10.0% estimate reflects reported use of an instrument with published evidence of validation in neonatal populations according to the study's predefined classification. The present findings indicate that previous availability or reported use of a validated scale does not necessarily correspond to current reported knowledge or reported use among healthcare professionals. Differences in dissemination, continuing education, or institutional implementation may contribute to this gap, although these factors were not directly assessed in the present study. Similar knowledge-to-practice gaps have been described among healthcare professionals in other settings (16, 20, 21).

The principal finding was the independent association between knowledge and reported use of a validated neonatal pain-assessment scale. Using the four-item knowledge score, each additional knowledge point was associated with approximately five-fold higher odds of reported scale use (adjusted OR, 4.90; 95% CI, 3.10–7.70; *p* < 0.001). However, recognition of a validated neonatal pain-assessment scale was itself one component of the knowledge score and was conceptually related to the primary outcome. The prespecified sensitivity analysis excluding this item showed that the association remained statistically significant, although substantially attenuated (adjusted OR, 1.93; 95% CI, 1.23–3.03; *p* = 0.004). This reduction in effect size suggests that the scale-recognition item contributed to the magnitude of the primary association and supports cautious interpretation of the four-item estimate. Model discrimination was lower in the reduced-score analysis (AUC, 0.745), with poorer calibration according to the Hosmer–Lemeshow test (*p* = 0.004). More importantly, the cross-sectional design does not establish that higher knowledge causes greater reported use of a validated neonatal pain-assessment scale.

Knowledge was also independently associated with good reported practice. In Model 2, each additional point on the reduced three-item knowledge score was associated with higher odds of good reported practice (adjusted OR, 2.15; 95% CI, 1.47–3.15; *p* < 0.001). Medical specialist status was also independently associated with good reported practice compared with nursing professionals (adjusted OR, 2.70; 95% CI, 1.30–5.61; *p* = 0.008). These associations should be interpreted as observational rather than causal. The cross-sectional design does not establish causality, and the observed associations should not be interpreted as evidence that increasing knowledge or specialist status would necessarily improve clinical practice.

The reported use of analgesics and non-pharmacological methods should also be interpreted cautiously. Paracetamol was the most frequently reported analgesic, while non-pharmacological methods were widely reported. Opioids were reported less frequently. Ibuprofen use warrants particular caution in neonates because its use in this population differs from its established use in older children and adults and is subject to age- and context-specific safety considerations. The present survey did not collect sufficient clinical information to determine the indications or appropriateness of reported ibuprofen use. Similarly, the limited information collected on tramadol use does not allow conclusions regarding its appropriateness in neonates.

More generally, these findings describe self-reported analgesic use and do not allow assessment of prescribing appropriateness because indication, dose, route, timing, gestational age, postnatal age, and patient-specific contraindications were not systematically collected. Accordingly, the reported frequencies should not be interpreted as evidence of either appropriate or inappropriate analgesic prescribing. Likewise, the reported use of breastfeeding, skin-to-skin contact, pacifier use, or containment should be understood as reported postoperative supportive/analgesic measures and not assumed to represent equivalent analgesic effects or to replace standardized pain assessment.

The institutional findings should also be interpreted as exploratory. Differences between institutions were observed across knowledge, assessment practices, and analgesic patterns. However, the institutional PCA and hierarchical clustering analyses were based on only ten institutions and should therefore be considered hypothesis-generating rather than evidence of stable or generalizable institutional phenotypes. Although hospital-level cluster-robust standard errors were used in the regression analyses, the small number of hospitals limits the precision with which between-institution variability can be characterized.

### 4.1 Strengths and limitations

This study has several strengths, including its multicenter design, inclusion of the ten hospitals providing pediatric surgical care in Kinshasa during the study period, and relatively large sample. The analytical strategy also distinguished cognitive knowledge from reported practices and included a prespecified sensitivity analysis to address potential overlap between scale recognition and the primary outcome.

Several limitations should be considered. First, practices were self-reported and therefore subject to recall, social-desirability, and misclassification biases. Second, the neonatal pain-assessment scales considered validated were supported by neonatal validation studies but were developed or validated in different pain contexts rather than specifically for postoperative neonatal pain; their reported recognition or use should therefore not be interpreted as evidence of use of a scale specifically validated for postoperative pain. Third, the study-specific questionnaire underwent expert content review and pretesting but was not formally validated as a measurement instrument. Similarly, the knowledge and good-practice scores were study-specific constructs and should not be interpreted as externally validated clinical or psychometric measures. Fourth, recognition of a validated neonatal pain-assessment scale overlapped conceptually with the primary outcome; however, the prespecified sensitivity analysis excluding this item yielded a persistent, although attenuated, association. Fifth, the cross-sectional design precludes causal inference, and residual non-response bias cannot be excluded despite the high response rate. Sixth, institutional PCA and hierarchical clustering were based on only ten hospitals and should be considered exploratory. Finally, Model 2 included 18 parameters for 89 outcome events (approximately 4.9 events per parameter), raising the possibility of overfitting and model instability; its estimates, particularly institution-specific effects, should therefore be interpreted cautiously.

## 5 Conclusion

Postoperative neonatal pain assessment was infrequently reported among healthcare professionals in the ten participating hospitals in Kinshasa, despite widespread recognition of neonatal pain. Higher knowledge was strongly associated with reported use of a validated neonatal pain-assessment scale, although the cross-sectional design and conceptual overlap between knowledge and scale recognition preclude causal interpretation. The findings also showed a substantial reported lack of dedicated training and limited reported use of validated neonatal pain-assessment scales.

These findings highlight the need to evaluate multidisciplinary training, institutional protocols, and implementation strategies in future intervention studies.

## Data Availability

The raw data supporting the conclusions of this article will be made available by the authors, without undue reservation.
