# Supplementary material for: Knowledge and practices regarding postoperative neonatal pain assessment among healthcare professionals in Kinshasa: a multicenter cross-sectional survey
Source: Front Pain Res (Lausanne). 2026 Oct 7;7:1968968. doi: 10.3389/fpain.2026.1968968 (PMC13683723; doi:10.3389/fpain.2026.1968968)
Supplement: Supplementary file 1 [file Datasheet1.pdf]

SUPPLEMENTARY FILE 1

English Version of the Study Questionnaire

Knowledge and Practices Regarding Postoperative Neonatal Pain Assessment in Kinshasa

Instructions: This questionnaire is anonymous. Please answer the questions according to your knowledge and usual practices.

I. PROFESSIONAL INFORMATION

1. In which institution do you work?

☐ Kinshasa University Clinics (CUK)
☐ CHU Renaissance
☐ Ngaliema Clinics
☐ Tshatshi General Hospital
☐ Kalembelembe Pediatric Hospital
☐ Monkole Hospital Center
☐ Ngaliema Medical Center
☐ Biamba Marie Mutombo Hospital (HBMM)
☐ Diamant Medical Center
☐ SOS Médecin de Nuit
☐ Other: ______________________

2. What is your profession?

☐ General practitioner
☐ Pediatrician
☐ Anesthesiologist-intensivist
☐ Surgeon
☐ Obstetrician-gynecologist
☐ Intern / resident in specialist training
☐ Medical trainee
☐ Nurse
☐ Anesthesia technician
☐ Nursing student
☐ Other: ______________________

3. How long have you been involved in the care of newborn infants?

☐ Less than 2 years
☐ 2–5 years
☐ 6–10 years
☐ More than 10 years

II. KNOWLEDGE OF NEONATAL PAIN

4. In your opinion, do newborn infants experience pain?

☐ More intensely than adults
☐ To the same extent as adults
☐ Less intensely than adults

☐ I don't know

5. The nociceptive system of a newborn infant is:

☐ Completely developed at birth
☐ Partially developed but functional
☐ Immature and ineffective
☐ I don't know

6. Which clinical signs do you associate with neonatal pain?
(Multiple responses possible)

☐ Specific facial expressions (grimacing, frowning)
☐ Changes in vital signs (HR, RR, SpO_2)
☐ Withdrawal movements or agitation
☐ High-pitched continuous crying
☐ Other: ______________________

7. Which neonatal pain-assessment scales do you know and feel able to use?
(Multiple responses possible)

☐ Neonatal Infant Pain Scale (NIPS)
☐ Premature Infant Pain Profile (PIPP)
☐ Échelle Douleur Inconfort Nouveau-Né (EDIN)
☐ Douleur Aiguë du Nouveau-né (DAN)
☐ Évaluation Enfant DouLeur (EVENDOL)
☐ Neonatal Facial Coding System (NFCS)
☐ COMFORT / COMFORT-B
☐ I do not know any of these pain scales

III. PAIN-ASSESSMENT PRACTICES

8. How often do you assess pain after surgery in a newborn infant?
(Multiple responses possible)

☐ At postoperative admission only
☐ At regular intervals (e.g., every 4 hours)
☐ According to the clinical condition
☐ Never

9. Have you received training in neonatal pain management?

☐ Yes
☐ No

10. Do you use a standardized neonatal pain-assessment tool?

☐ Yes
☐ No

IV. BARRIERS TO PAIN ASSESSMENT

11. What are the main challenges you encounter when assessing neonatal pain?
(Multiple responses possible)

☐ Lack of specific training
☐ Lack of locally validated tools

☐ Difficulties in clinical interpretation
☐ High workload
☐ We do not assess neonatal pain
☐ Other: ______________________

V. PHARMACOLOGICAL MANAGEMENT

12. Which analgesics do you most frequently administer to newborn infants?
(Multiple responses possible)
☐ Paracetamol
☐ Ibuprofen
☐ Ketoprofen
☐ Morphine
☐ Fentanyl
☐ Sufentanil
☐ Remifentanil
☐ Tramadol
☐ Nalbuphine
☐ Ketamine
☐ Clonidine
☐ Midazolam
☐ Amitriptyline
☐ Methadone
☐ Other: ______________________

13. What do you base your choice of analgesic treatment on?
(Multiple responses possible)
☐ Institutional protocols
☐ International recommendations
☐ Clinical judgment
☐ Medication availability
☐ Other: ______________________

14. What are your reasons for reluctance to use analgesics in newborn infants?
(Multiple responses possible)
☐ No reluctance
☐ Fear of adverse effects
☐ I do not know the dosage of analgesics in newborn infants
☐ Scientific uncertainty
☐ Institutional pressure
☐ Other: ______________________

VI. NON-PHARMACOLOGICAL METHODS

15. Do you use non-pharmacological methods to relieve neonatal pain?
☐ Yes
☐ No

16. If yes, which methods do you use?

(Multiple responses possible)

☐ Breastfeeding
☐ Skin-to-skin contact
☐ Pacifier
☐ Containment
☐ Other: ______________________

VII. GENERAL CHALLENGES IN PAIN MANAGEMENT

17. What are the main challenges you encounter in the assessment and management of neonatal pain?

(Multiple responses possible)

☐ Lack of specific training
☐ Lack of locally validated tools
☐ Absence of written protocols
☐ Difficulties in clinical interpretation
☐ High workload and insufficient time to assess pain
☐ Lack of medications
☐ Failure to recognize neonatal pain
☐ Other: ______________________

VIII. SATISFACTION

18. Are you satisfied with this survey?

☐ Yes
☐ No

IX. POSTOPERATIVE NEONATAL PAIN MANAGEMENT PRACTICES

19. Is a written protocol or guideline for neonatal pain assessment and management available in your department?

☐ Yes
☐ No
☐ Don't know

20. In your usual practice, do you systematically document neonatal pain assessment in the patient's medical or nursing record?

☐ Yes
☐ No
